# Application of Ultrasound Localization Microscopy in Evaluating the Type 2 Diabetes Progression

**DOI:** 10.34133/cbsystems.0117

**Published:** 2025-03-10

**Authors:** Tao Zhang, Jipeng Yan, Xinhuan Zhou, Bihan Wu, Chao Zhang, Mengxing Tang, Pintong Huang

**Affiliations:** ^1^ Department of Ultrasound in Medicine, The Second Affiliated Hospital of Zhejiang University School of Medicine, Hangzhou, Zhejiang 310000, China.; ^2^ Research Center of Ultrasound in Medicine and Biomedical Engineering, The Second Affiliated Hospital of Zhejiang University School of Medicine, Hangzhou, Zhejiang 310000, China.; ^3^ Ultrasound Lab for Imaging and Sensing, Department of Bioengineering, Imperial College London, London, UK.; ^4^ Research Center for Life Science and Human Health, Binjiang Institute of Zhejiang University, Hangzhou, Zhejiang 310009, China.

## Abstract

Type 2 diabetes is considered as a chronic inflammatory disease in which the dense microvasculature reorganizes with disease progression and is highly correlated with β cell mass and islet function. In this study, we constructed rat models of type 2 diabetes and used ultrasound localization microscopy (ULM) imaging to noninvasively map the pancreatic microvasculature at microscopy resolution in vivo to reflect β cell loss and islet function deterioration, and evaluate the efficacy after anti-cytokine immunotherapy. It was unveiled that ULM morphological and hemodynamic parameters have a strong link with β cell loss and deterioration of pancreatic islet function. This correlation aligns with the observed pathological alterations in the microvessels of islet and demonstrated that ULM can effectively mirror the functionality of β cells during rapid fluctuations in blood glucose levels by observing changes in mean velocity. Furthermore, it was revealed that treatment with anti-cytokine immunotherapy enhances the function and health of β cells by restoring the microvascular environment. Remarkable improvements in vessel morphology (measured by fractal dimension) and hemodynamics (indicated by mean velocity and vessel density) were noted following the anti-cytokine immunotherapy, signifying a significant enhancement at the treatment’s conclusion (*P* < 0.05). These observations suggested that ULM technology holds promise as a visible and efficient tool for monitoring the effectiveness of anti-cytokine immunotherapy in managing type 2 diabetes. Pancreatic microvessel-based ULM may serve as a novel noninvasive method to assess β cells, providing a valuable clinical tool for tracking the progression of type 2 diabetes.

## Introduction

Type 2 diabetes is viewed as an autoinflammatory disease [1]. Long-term chronic inflammation will lead to fibrosis, collagen deposition, and functional vascular structure and function disorder of pancreatic islets, and then cause β cell dysfunction [2]. Within the scope of diabetes mellitus, the utilization of noninvasive imaging presents an avenue to figure out a myriad of questions. This approach enables an in-depth investigation into the intricate mechanisms that regulate metabolism, identifying precise outcomes where cellular or metabolic abnormalities indicate or potentially forecast the onset of disease [3,4]. Therefore, it is crucial to develop new methods to diagnose and characterize β cell function and mass through noninvasive imaging to the study of diabetes in biotechnology area.

However, the imaging of β cells poses a notable challenge due to the intricate biological and technological aspects involved. This challenge is rooted in the complex and varied structure and distribution of pancreatic islets within the pancreas, coupled with the dynamic and multifaceted nature of their metabolic function [5,6]. Notably, pancreatic islets are highly vascularized structures, constituting approximately 10% to 20% of the pancreatic blood volume [7]. Variations in islet blood flow may occur throughout the progression of insulitis and diabetes, as well as in responding to acute glycemic variations, closely tied to the overall pancreatic blood flow [8]. However, existing in vivo imaging technologies, including functional magnetic resonance imaging (MRI), Doppler ultrasound, and contrast enhanced ultrasound (CEUS), cannot monitor pancreatic microvascular morphology and hemodynamics in deep tissue due to their limited resolution and sensitivity to slow flow in these vessels.

Ultrasound localization microscopy (ULM) imaging, the acoustic counterpart of optical super-resolution, provides both a spatial and a temporal dimension by localizing and tracking microbubbles [9–11], whereby disease progression can be monitored longitudinally and is being applied to the imaging of brain, tumor, and other microvascular-related diseases [12–14]. Ultrafast ultrasound can benefit the microbubble tracking accuracy but is not always available in the commercial ultrasound system. Motion model-based tracking methods have been designed to achieve ULM with low-frame-rate acquisition, making ULM closer to clinic application [11,15]. Therefore, considering the changes of pancreatic microvascular morphology and hemodynamics in diabetes patients, we hypothesis that ULM imaging of pancreatic blood flow and the changes of these measurements over time will provide a noninvasive method to diagnose and track the state of pancreatic islets through the progress of type 2 diabetes.

In this study, we have attempted to provide quantitative information on pancreatic microvascular morphology and hemodynamics in their intact, authentic, physiological environment through ULM technology as predictive markers for type 2 diabetes progression and anti-cytokine immunotherapy efficacy. We compared these potential changes in ULM through histological analysis and by conducting assessment of biochemical indicators as well as monitoring efficacy of anti-cytokine immunotherapy. ULM imaging technology can achieve visualization based on microvascular function, supplementing the vacancy of the existing clinical technology in the evaluation of microvascular morphology. Thus, ULM technology provides a novel in vivo imaging paradigm for the identification, diagnosis, and monitoring of type 2 diabetes progression in patients.

## Methods

### Animal model

Male Sprague–Dawley rats (SD rats), 8 weeks old, were provided by Zhejiang Academy of Medical Sciences. Following acclimatization for 1 week, rats were fed with high-fed diet for another 2 weeks to set up the IR model. Following overnight fasting, it was attempted to intravenously inject with a low dose (35 mg/kg) of streptozotocin obtained from Sigma-Aldrich (St. Louis, MO, USA). One week following streptozotocin injection, FPG levels were tested and the rats above 300 mg/dl were considered diabetics [16].

### Ethical approval

Animal experiments received approval from the Institutional Animal Care and Use Committee at the Zhejiang Center of Laboratory Animals (approval no. ZJCLA-IACUC-20060009).

### Image acquisition

B-mode and CEUS images were acquired by a commercial ultrasound system (ZONARE ZS3, Mindary, China) and an L30-8 linear probe. For CEUS imaging, 0.12 μl ultrasound contrast agent (Sonazoid, GE Healthcare, Amersham, UK) was continuously injected (0.03 μl/min) via the tail vein for 4 min to maintain a stable concentration in the bloodstream. The microbubbles underwent circulation within the animal for an approximate duration of 120 s, facilitating their attainment of a relative steady state of the systemic distribution. The microbubble dose injected in the rats was 0.40 μl/kg, which was significantly higher than the clinical recommendation (0.12 μl/kg) [17], but relatively less than the prescribed safe dose (0.60 μl/kg) for Sonazoid [18]. The data were then acquired for 20 s. Acquisition settings used were as follows: mechanical index (MI) 0.21, frequency 15 MHz, imaging depth 1.0 cm, and dynamic range 60/70, with an acquisition rate of 55 frames per second. B-mode and CEUS images were placed in 2 columns and saved as “.avi” videos with pixel dimension of 32 μm × 32 μm from the system.

### ULM imaging

**Fig. 1.**
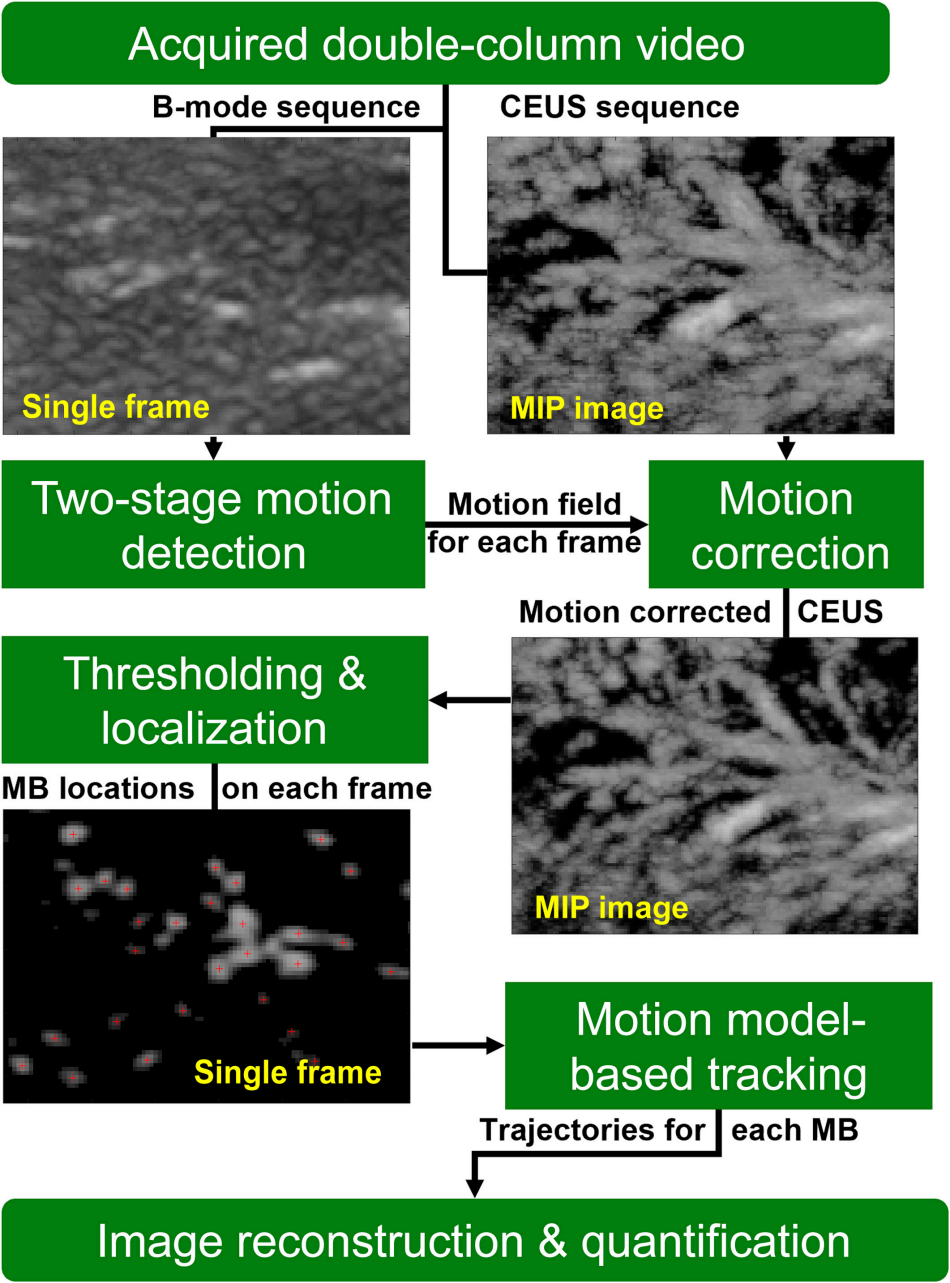
Diagram of ULM processing.

The ULM imaging was conducted in Matlab software (2021a, Mathworks, USA) by offline postprocessing of the videos. As shown in Fig. 1, B-mode and CEUS images were cropped from each frame correspondingly by masking out the region of pancreas in the B-mode image. Tissue motions were estimated between a moving and a reference B-mode images by a 2-stage image registration algorithm [11], where the B-spline-based registration method was used to detect the nonrigid local deformation after using affine transformation to detect the global motion. Estimated tissue motion fields were reversed and then applied to the respective CEUS image to guarantee that localized microbubbles were accumulated in right positions in the ULM processing. Each CEUS sequence was then normalized by its maximum. A framework proposed in a previous report was adopted for super-localization and microbubble tracking. The code of the framework can be found at https://github.com/JipengYan1995/ImperialSRUS. Each CEUS frame and the point spread function (PSF) estimated from the data were interpolated to a map with 4 μm × 4 μm pixels. Microbubbles were localized by the peaks on the cross-correlation coefficient map obtained by normalized cross-correlation between each CEUS image and the estimated PSF (demonstration of localization can be found in Movie S1).

To obtain the blood velocities and the trajectories of bubble movements, a cost function is defined as $p^{-1}$ for pairing bubbles, where *p* is a probability estimated by a linear Kalman motion model. Microbubbles in 2 adjacent frames were paired by finding total minimum of the cost function via a graph-based assignment and disappearing as newly appearing behaviors of microbubbles were also considered. Only microbubbles that appeared no less than 3 frames were kept for plotting ULM images and calculating metric parameters (demonstration of blood flow can be found in Movie S2).

ULM images were plotted by accumulation or vector mean of microbubble trajectories for density maps or flow maps with 2-dimensional (2D) Gaussian or disk smoothing to represent the localization uncertainty of ULM.

Tortuosity was given as the ratio of length of an MB trajectory to distance between its 2 ends, which helped to avoid effects of errors in vessel segmentation on quantifying curvature of vessels. Fractal dimension, vessel density, and mean vessel diameter [19] were calculated from the binarized ULM density map to quantify vasculature. Fractal dimension quantified the complexity of the vasculature as a ratio of change in details to the change in scale. Smaller fractal number demonstrated vessel branch more regularly. Mean velocity was calculated by averaging values on ULM velocity maps. Vessel density was calculated by the ratio of area covered by vessel on binarized ULM density map to the area of region of interest. Center lines of vessels were detected on binarized ULM density maps, and the vessel diameters were calculated by double the distance between the center line and its nearest boundary on binarized ULM density maps.

### Biochemical assessment

The blood was collected from the retrobulbar vein, and the serum collected in tubes was then centrifuged at 1,000 g, 10 min, 4 °C and subsequently used for biochemical analysis. The expression levels of interleukin-1β (IL-1β), IL-6, and tumor necrosis factor-α (TNF-α), which were all obtained from Thermo Fisher Scientific (Waltham, USA), were quantified on the basis of instructions outlined by the manufacturer.

### Immunofluorescent staining

Immunofluorescence is used to detect antibodies specific for a single target protein and is often visualized by conjugating a fluorophore directly to the primary antibody [20]. For immunofluorescent staining, it was attempted to deparaffinize and incubate the paraffin-embedded tissue sections particularly with primary antibodies against insulin obtained from Sigma-Aldrich or Ki67 obtained from Beijing Solarbio Science & Technology Co. Ltd. (Beijing, China) overnight at 4 °C. Following phosphate-buffered saline (PBS) rinsing, it was attempted to incubate the sections with Alexa Fluor-conjugated secondary antibody (Cell Signaling Technology, USA) or Cy3-conjugated secondary antibody (Beijing Solarbio Science & Technology Co. Ltd.). Nuclear counterstaining was achieved using 4′,6-diamidino-2-phenylindole (DAPI), and the visualization of sections was undertaken utilizing an epifluorescence microscope obtained from Nikon (Tokyo, Japan). After insulin staining, it was attempted to add and co-incubate TUNEL (terminal deoxynucleotidyl transferase–mediated deoxyuridine triphosphate nick end labeling) dye, which was obtained from Beyotime Biotechnology Co. Ltd. (Shanghai, China), with tissue paraffin slices for 120 min. Subsequently, DAPI staining was undertaken to label cell nuclei. Quantification of positive areas and numbers was conducted utilizing the Analyze Particle function in ImageJ (version 2.0.0-rc-69/1.52i). To enhance the visibility of fluorescent signals, adjustments to contrast and brightness were applied to some fluorescence images using Microsoft PowerPoint.

### Vessel labeling

Under anesthesia, intravenous injection of 500 μl of fluorescein isothiocyanate (FITC)-conjugated tomato lectin (*Lycopersicon esculentum* lectin), which was obtained from Vector Labs (Burlingame, CA, USA), to rats was undertaken. Following isolation of the pancreas, it was attempted to fix tissues in 4% paraformaldehyde on ice particularly for 1 h and cryoprotect in 30% sucrose overnight or until the tissue sank. The embedding of lectin-infused pancreas in optimal cutting temperature compound (OCT) medium was undertaken, followed by freezing in cryomolds, and sectioning at 10- to 20-μm thickness. These sections were imaged at 595-nm excitation through an LSM800 confocal microscope obtained from Zeiss. Confocal fluorescence microscopy is an effective tool for quantitative measurements of cells and tissues [21]. Three-dimensional reconstructions were generated by combining 10 *Z*-axial confocal layers with 8- to 10-μm focus steps, resulting in a total section thickness of approximately 80 to 100 μm.

### Flow cytometry

To analyze the intracellular staining for interferon-γ (IFN-γ) and IL-17a, cells were stimulated with Cell Stimulation Cocktail (eBioscience, CA, USA) for 6 h at 37 °C. Activated cells were then incubated with antibodies specific for CD45 (BioLegend, San Diego, CA), CD4 (Thermo Fisher Scientific), IFN-γ (Thermo Fisher Scientific), and IL-17a (Thermo Fisher Scientific) at 4 °C for 30 min before acquisition. For staining T_reg_ (T regulatory), cells were incubated with antibodies specific for CD45, CD4, and CD25 (Thermo Fisher Scientific), and then fixed and permeabilized with Fixation/Permeabilization Buffer (Invitrogen). After that, cells were incubated with Foxp3 (BioLegend). Flow cytometric analysis was undertaken through a CytoFLEX flow cytometer and the CytExpert software program, which were both obtained from Beckman Coulter.

### Statistical analysis

The statistical analysis was undertaken through R 4.2.2 software that was developed by R Foundation. For comparable analysis particularly between groups, the Kruskal–Wallis test or paired *t* test was utilized for abnormally and normally distributed data, respectively. In instances where multiple measurements were taken per mouse, a mixed-effects model was employed and implemented through SPSS 26.0 software developed by IBM (Armonk, NY, USA). A *P* threshold below 0.05 was suggestive of statistical significance.

## Results

### Quantification of resolution of ULM image

**Fig. 2.**
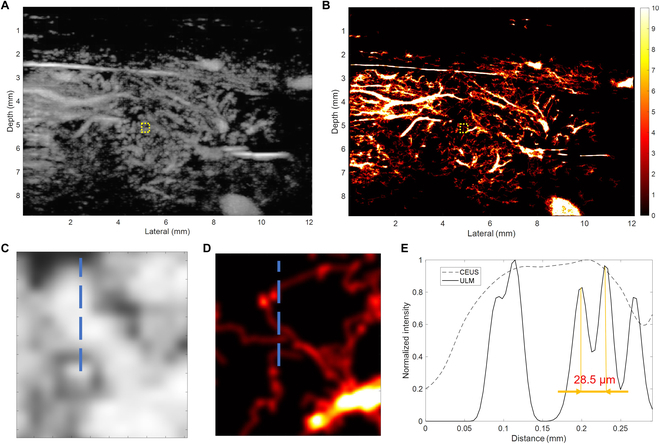
The resolution improvement and quantification of the ULM image. (A) CEUS MIP image and (B) ULM density image of pancreas. (C) Magnified CEUS MIP and (D) ULM density images of the same region within the yellow boxes. (E) Vessel intensity profiles along the blue line on the magnified CEUS MIP and ULM images.

The ULM imaging of the rat pancreas was first performed. The resolution of CEUS maximal intensity projection (MIP) imaging (Fig. 2A) was significantly improved by ULM imaging (Fig. 2B). From the image intensity cross-sectional profiles (Fig. 2C to E), microvessels that were 28.5 μm apart can be visually resolved in the ULM image but are not separable in the CEUS MIP image. ULM images have higher resolution than traditional CEUS images. By tracking MBs, they can display the dynamic and morphological information in small blood vessels that cannot be obtained by traditional ultrasound, and accurately quantify this information. Then, these quantitative indicators can be used to evaluate acute blood glucose changes and functional changes of β cells at different stages of the disease.

### Type 2 diabetes progression and ULM monitoring

**Fig. 3.**
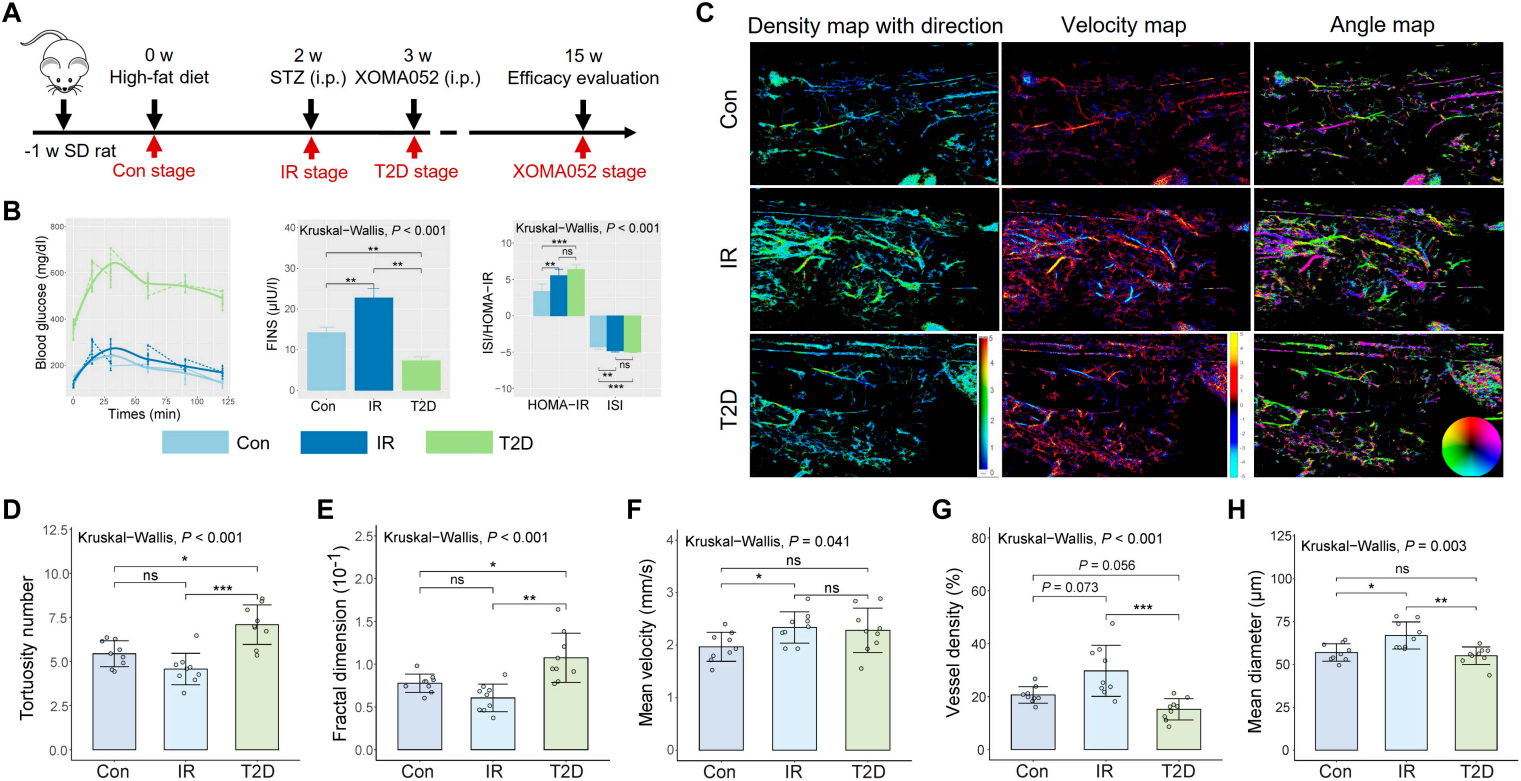
Longitudinal study of real-time ULM imaging in vivo using a type 2 diabetes model. (A) Study design. (B) The intraperitoneal glucose tolerance test (ipGTT), fasting serum insulin (FINS), homeostatic model assessment of insulin resistance (HOMA-IR) and insulin resistance index (ISI) of T2D progression. (C) ULM imaging in the progression of T2D, including density map with direction, velocity map, and angle map. ULM values in the progression of T2D, (D) tortuosity number, (E) fractal dimension, (F) mean velocity, (G) vessel density and (H) mean diameter. (*n* = 9). * = *P* < 0.05, ** = *P* < 0.01, *** = *P* < 0.001, ns = nonsignificant.

A rat model that replicated the natural history and metabolic characteristic of human type 2 diabetes was first established (Fig. 3A). As shown in Fig. 3B, after 2 weeks of high-fed diet feeding, rats remained euglycemic due to compensatory insulin secretion, but with impaired insulin resistance index (ISI) and the homeostatic model assessment of insulin resistance (HOMA-IR), mimicking a pre-symptomatic disease state (insulin resistant, IR stage). One week after streptozotocin (STZ) injection, intraperitoneal glucose tolerance test (IPGTT) showed a significant increase of blood glucose screening, while ISI and HOMA-IR remain relatively constant throughout the onset of the disease.

ULM images in rats during type 2 diabetes progression are shown in Fig. 3C. To provide microvascular structure in pancreas and, in turn, variations in islet hemodynamic information, we performed ULM image in rats during type 2 diabetes progression. The bubble localization positions can indeed be accumulated to build an anatomical ULM image to reconstruct functional microvessels of pancreas in vivo noninvasively over time, and its morphological measurements (tortuosity and fractal dimension) can be quantified. Tortuosity describes the degree of curvature of a vessel (i.e., how much the vessels twist and turn), and fractal dimension describes how the vascular pattern fills a 2D space and is thus a measure of complexity or pathological changes of the vascular network.

In the normal (control) and pre-symptomatic (IR) stage, the vascular structure remained orderly, reflected in the measurements of tortuosity (Fig. 3D) and fractal dimension (Fig. 3E), which increased dramatically when it progressed to the type 2 diabetes stage, indicating that the microvessels turned twisting and disordered. More interestingly, tracking these localization positions generates a trajectory that provides each microbubble with an instantaneous 2D speed vector, revealing the hemodynamics of the vessels. The mean velocity was slightly increased in the IR stage compared to the control group (2.49 ± 0.29 versus 1.97 ± 0.28 mm/s, *P* < 0.05) (Fig. 3F). Previous research showed that pancreatic islet vasculature adapts to insulin resistance through dilation and not angiogenesis [22]. Consistent with this report, vessel density (Fig. 3G) and mean diameter (Fig. 3H) increased in the IR stage and turned into a sharp decline during the type 2 diabetes phase. The differences of the above measurements ​​at different stages of type 2 diabetes indicate that ULM imaging of pancreatic microvessels can reflect the hemodynamic and morphological characteristics of pancreatic microvessels and has potential for sequential monitoring of type 2 diabetes progression in vivo.

### Pathological examination

**Fig. 4.**
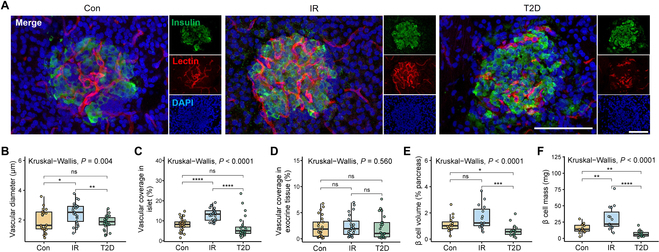
Changes in islet microvascular morphology in SD rats. (A) Representative maximum-projection confocal image between 8- to 10-μm depth of pancreas section from SD rat infused with FITC-conjugated tomato insulin (green), lectin (red), and DAPI (blue) immunofluorescence staining of pancreatic islets in different stages of T2D. Scale bar, 20 µm. (B) Vascular diameter in different disease stages of T2D. (C) Vascular coverage in islet and (D) in exocrine tissue in the T2D progression (*n* = 25 rat islets from 5 rats). (E) β cell volume and (F) β cell mass in different disease stages of T2D (*n* = 15 panoramic scans of pancreas from 5 rats). *P < 0.05, **P < 0.01, ***P < 0.001, ns = nonsignificant.

We next tested whether the observed changes in ULM measurements in rats are consistent with pathological changes in the islet microvascular system associated with type 2 diabetes progression. We visualized the islet microvessels of SD rats in different stages of type 2 diabetes by intravital labeling with lectin-FITC. This methodology takes advantage of intravascular labeling that visualizes only functional vessels and, when combined with optical sectioning, allows for 3D reconstruction of the islet angioarchitecture. This 3D reconstruction revealed that in the early stage of the disease, the microvessels in the islets are continuous and smooth; in the type 2 diabetes stage, the morphology of microvessels changes, showing discontinuity and distortion (Fig. 4A), which is consistent with the results obtained in ULM morphological measurements. Within the islets, we observed a significant increase in vessel diameter in the IR stage (2.39 ± 0.73 μm) compared to the control (1.93 ± 0.72 μm) and type 2 diabetes stage (1.84 ± 0.45 μm) (Fig. 4B). Meanwhile, we observed an increased area of vasculature coverage within the islets in the IR stage compared to others (Fig. 4C), with no significant change in the exocrine tissue (Fig. 4D). As the model progresses to type 2 diabetes, the consistent changes of ULM measurements on islet blood flow dynamics, but not on exocrine blood flow, further support the idea that these whole-pancreas ULM measurements can reflect changes in the dynamics of islet blood flow at different stages of type 2 diabetes.

As well as the microvascular changes, we also quantified β cell volume, which is an important factor in the development of type 2 diabetes [23]. The results showed that β cell volume was significantly reduced in the type 2 diabetes stage compared to the IR stage (*P* < 0.001) and slightly reduced compared to the control group, but there was no statistical difference (*P* = 0.054) (Fig. 4E). Also, the β cell mass is based on pancreatic weight in each stage, with statistically significant reductions in the type 2 diabetes stage compared with the other stages (Fig. 4F), and consistent with the change of vessel density and mean diameter fluctuation.

### ULM imaging in vivo islet response to glucose stimulation

**Fig. 5.**
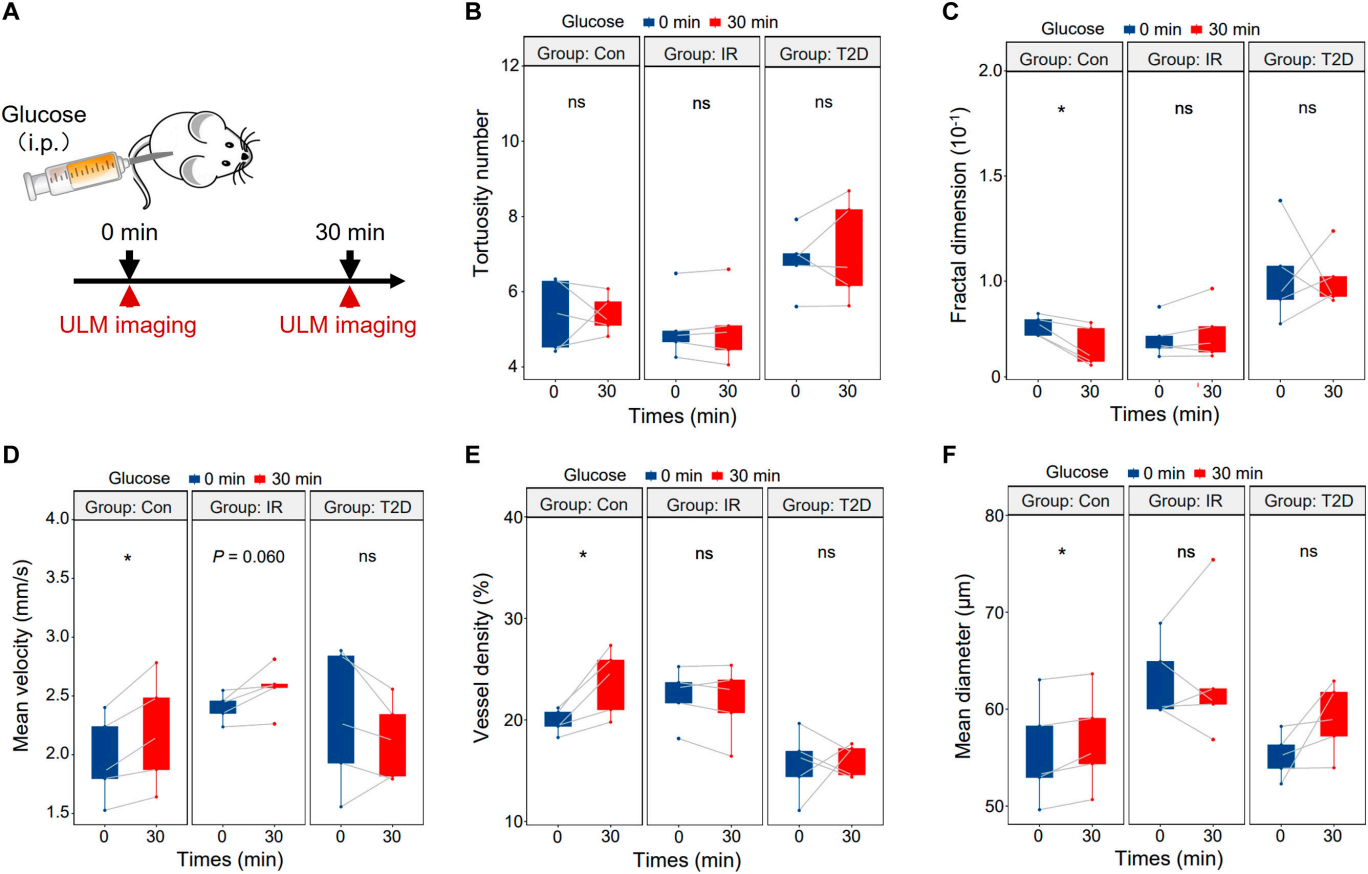
Noninvasive β cell function evaluation in vivo after glucose stimulation. (A) Study design. Measurements of (B) tortuosity number, (C) fractal dimension, (D) mean velocity, (E) vessel density, and (F) mean diameter in the control, IR, and T2D groups (*n* = 5). *P < 0.05, ns = nonsignificant.

Prior studies have shown increases in islet blood flow velocity in response to acute increases in the blood glucose levels to reflect the β cell function under acute blood glucose changes [24–26]. Similar to these reports, as shown in Fig. 5, we observed a significant increase in mean velocity, vessel density, and mean diameter in the control group 30 min after glucose delivery during IPGTT, accompanied by an acute increase in blood glucose [27]. In the IR group, the compensation for acute glucose change was only manifested as a slight increase in mean velocity, which was not statistically significant (*P* = 0.060). In these animals, there was no significant correlation between vessel density, mean diameter, and the degree of glucose intolerance. In contrast, no significant changes were found in the above measurements during type 2 diabetes group after glucose stimulation, indicating that the morphology and function of pancreatic vessels did not change.

### ULM imaging in evaluating efficacy of anti-cytokine immunotherapy

**Fig. 6.**
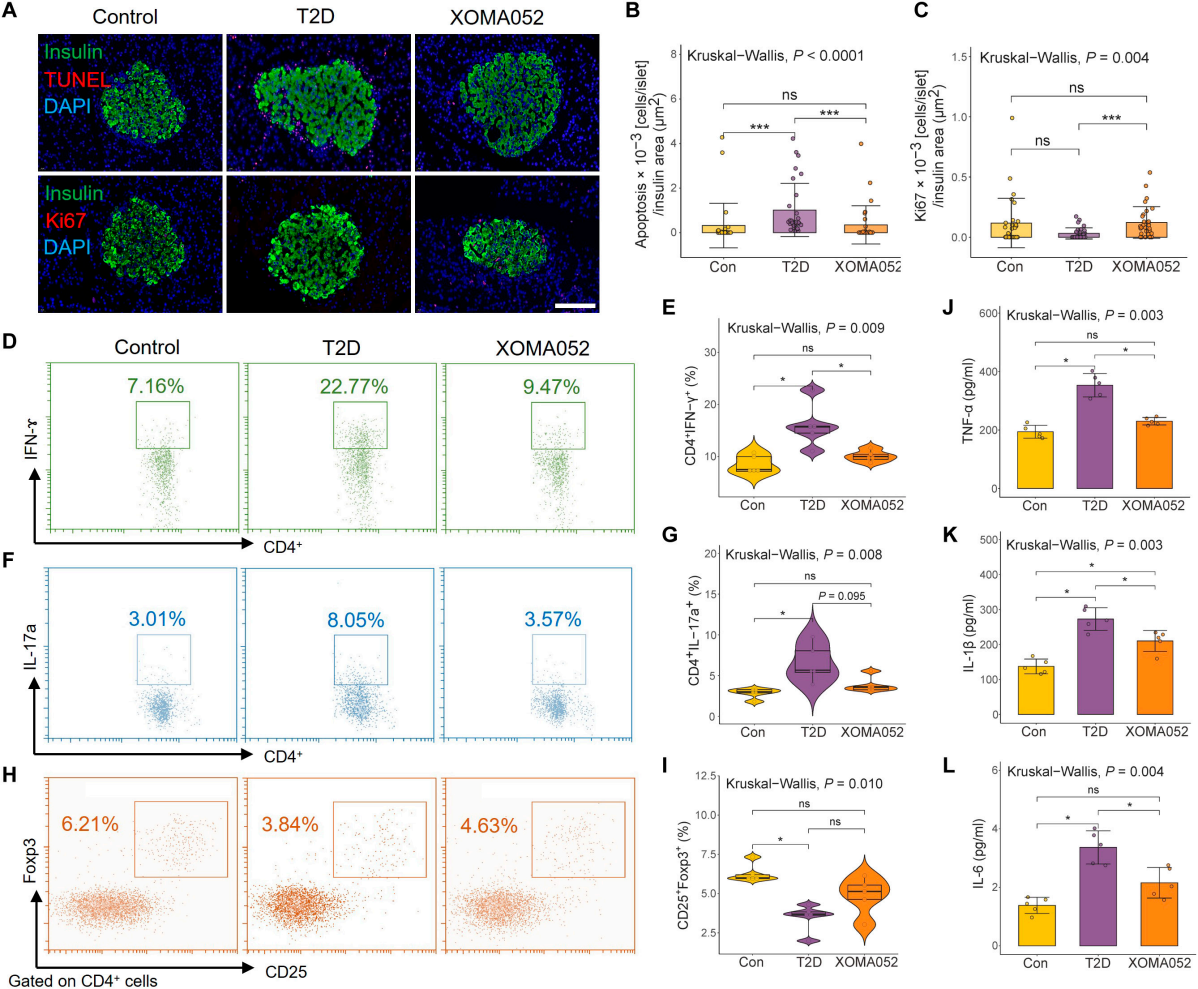
XOMA052 promotes β cell proliferation and evaluates the efficacy of anti-cytokine immunotherapy. (A) Fluorescence staining image of TUNEL and Ki67 of pancreatic islets in SD rats. Scaler bar, 100 μm. Corresponding positive cell number of (B) TUNEL and (C) Ki67 in fluorescence staining image (*n* = 30 rat islets from 5 rats). (D and E) Representative flow cytometry plots showing the CD4^+^ IFN-ɤ^+^ T cells in pancreatic islets and quantification. (F and G) Representative flow cytometry plots showing the CD4^+^ IL-17a^+^ T cells in pancreatic islets and quantification. (H and I) Representative flow cytometry plots showing the CD4^+^ CD25^+^ Foxp3^+^ T cells in pancreatic islets and quantification. Cytokine levels of (J) TNF-α, (K) IL-1β, and (L) IL-6 in sera from rats in different stages (*n* = 5). *P < 0.05, ***P < 0.001, ns = nonsignificant.

The introduction of monoclonal antibody that targets IL-1β has achieved success in the treatment of chronic immune-inflammatory diseases such as psoriasis, arthritis, or type 2 diabetes [28–30]. After XOMA052 anti-cytokine immunotherapy, β cell mass was improved by reducing apoptosis and promoting proliferation, which was reflected in the TUNEL and Ki67 stain (Fig. 6A to C). We further examined the anti-inflammatory effect of XOMA052. XOMA052 treatment can substantially reduce IFN-γ (Fig. 6D and E) and slightly down-regulate the production of IL-17a (Fig. 6F and G) from peripheral blood to alleviate chronic inflammation, but has no significant effect on the proportion of T_reg_ cells (Fig. 6H and I). These data indicated that T cells in type 2 diabetes rats are naturally skewed toward proinflammatory subsets and can alleviate chronic inflammation in type 2 diabetes by reducing inflammatory cytokines, such as TNF-α, IL-1β, and IL-6 (Fig. 6J to L).

**Fig. 7.**
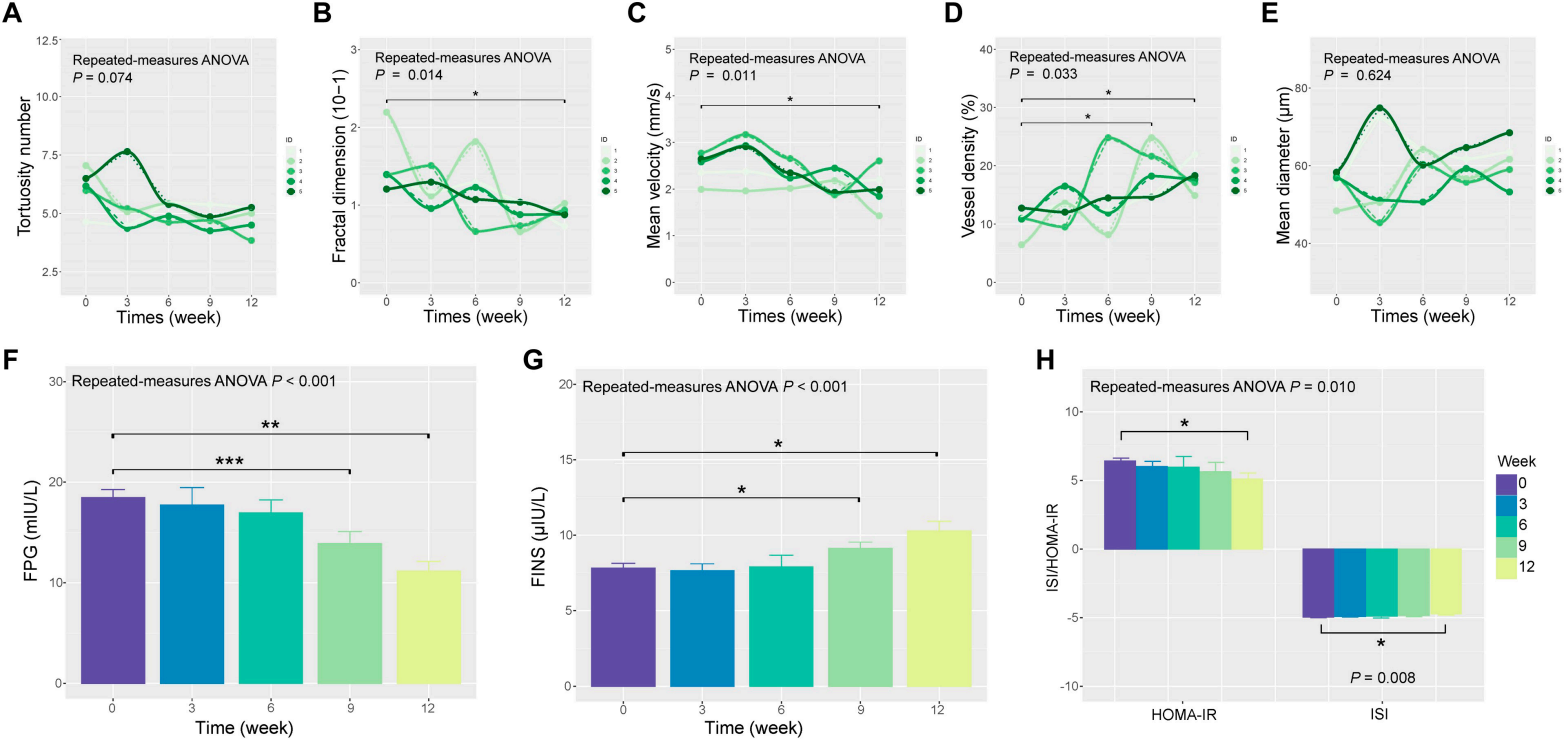
ULM imaging in efficacy evaluation of anti-cytokine immunotherapy in type 2 diabetes. (A) The tortuosity number, (B) fractal dimension, (C) mean velocity, (D) vessel density, (E) mean diameter, (F) fast plasma glucose (FPG), (G) fasting serum insulin (FINS), (H) homeostatic model assessment of insulin resistance (HOMA-IR), and insulin resistance index (ISI) of T2D rats evaluated at 0, 3, 6, 9, and 12 weeks after anti-inflammatory treatment with XOMA052 (*n* = 5). *P < 0.05, **P < 0.01, ***P < 0.001.

To further illustrate the role of anti-cytokine immunotherapy in reversing β cell mass and its subsequent impact on microvascular flow, we sequentially monitored the course of treatment by ULM (Fig. 7A to E). We found that the fractal dimension, mean velocity, and vessel density were significantly relieved (*P* < 0.05) at the end of the treatment course. Moreover, the fast plasma glucose (FPG), fasting serum insulin (FINS), HOMA-IR, and ISI (Fig. 7F to H) also showed better efficacy after 12 weeks through continuous monitoring.

## Discussion

It was attempted to conduct a validation study to figure out the feasibility of utilizing ULM (ultrafast laser microscopy) imaging for noninvasive monitoring and tracking of type 2 diabetes progression and its therapeutic reversal particularly in preclinical models. This innovative approach promoted visualizing and analyzing intricate microvascular morphological and hemodynamic parameters within the pancreas at a microscopic scale. In this study, the structure of pancreatic microvessels can be visualized and further quantified by measuring tortuosity and fractal dimension via ULM imaging. We demonstrated that the microvascular morphological changes of pancreas are highly correlated with progressive inflammatory infiltration and proinflammatory cytokine production, including TNF-α, IL-1β, and IL-6, which could lead to β cell decline and hyperglycemia with age. The qualitatively similar results achieved in all type 2 diabetes models highlight the power of ULM in application of microvascular-related diseases.

The ULM processing under study was designed to be generally usable and efficient. B-spline-based nonrigid image registration has been established with codes available online. Compared to deconvolution [11,31,32], normalized cross-correlation [33] is more efficient and can provide comparable localization accuracy at the microbubble concentration used in this study. Microbubbles that can be tracked by the Hungarian method or Kuhn–Munkres algorithm with ultrafast ultrasound [11,34] might not be tracked at the frame rate of our used commercial ultrasound system. Motion models were implemented to track microbubbles with data association [32,35] or graph-based assignment [36]. While the data association pairs microbubbles in multiple frames, the graph-based assignment pairs microbubbles in 2 consecutive frames and thus was used in our ULM processing pipeline for its high efficiency.

Existing approaches to monitor β cell mass is usually achieved by specifically labeling β cell volume, which has proven problematic in in vivo monitoring, potentially due to the low proportion of the overall pancreas volume and exocrine-originating background signal [37]. Importantly, pancreatic islets are highly vascularized and correlated with islet blood flow in euglycemic SD rats (data based on 36 controlled experiments, *P* < 0.001). Changes in the islet microvasculature, but not exocrine vasculature, have also been observed in animal models of type 2 diabetes and in human donors with type 2 diabetes [38,39]. Three-dimensional fluorescence reconstruction of the functional microvascular structure change in the type 2 diabetes stage correlates with loss of the β cells and the deterioration of pancreatic islet function. Also, the noninvasive ULM measurements we performed detected changes in blood flow dynamics, especially changes in vessel density and mean diameter, consistent with our observations of islet microvascular remodeling, and we did not observe significant changes in the exocrine vascular, indicating the correlate measured changes in the vessel density and mean diameter across the pancreas changes in the β cell mass, and assess type 2 diabetes progression in the models examined.

Based on the well-perfused islet compared to the poorly perfused exocrine, we inferred that blood flow dynamics of pancreas reflect islet blood flow dynamics. Initially, insulin resistance leads to a compensatory increase in β cell mass that can delay or even prevent the development of type 2 diabetes [40,41]. Effectively coping with states, slightly increased mean velocity (*P* = 0.060) represents a compensatory of β cell function, thereby maintaining euglycemia, until hyperglycemia supervenes. It was consistent with the results of an autopsy report of 124 people in the Mayo Clinic [42]. The adaptive hypervascularization was very important for maintaining the survival of β cells [43]. During this period, β cell responsiveness to acute blood glucose changes was reduced, indicating impaired β cell function. In obese type 2 diabetes stage, loss of pancreatic β cell mass and failure of the remaining β cell function cannot provide sufficient insulin to meet the needs of the body. ULM measurements manifested as a decrease in vessel density and mean diameter, and the compensatory increase in mean velocity disappears in the case of acute blood glucose changes. Thus, accurate measurement of vascularization in vivo to evaluate functional and mass changes in β cells will greatly enhance our understanding of type 2 diabetes progression, and the choice of diabetes treatment, making it a major research focus.

The anti-cytokine immunotherapy of type 2 diabetes is a new treatment method in recent years, especially blocking IL-1β in the process of impaired insulin secretion and insulin resistance, and can be used as a potential therapeutic target to reverse the quality and function of β cells [44–46]. We found that at the end of treatment, XOMA052 was able to alleviate chronic inflammation in type 2 diabetes by down-regulating the production of IFN-γ and IL-17a in peripheral blood, reducing inflammatory cytokines TNF-α, IL-1β, and IL-6, and reducing β cell apoptosis and promoting proliferation. Based on the sequential ULM measurement monitoring after XOMA052 treatment, the fractal dimension, mean velocity, vessel density, FPG, FINS, HOMA-IR, and ISI were all alleviated at the end of the treatment course. Therefore, we speculate that anti-cytokine immunotherapy can improve β cell function and mass by reversing microvascular status. ULM technology is anticipated to emerge as a visual and impactful method for monitoring anti-cytokine immunotherapy in type 2 diabetes.

Our research still has certain limitations. Reconstructed vessels might not be fully saturated with the limited acquisition duration in this study, which makes measurements of vessel morphology not fully independent from hemodynamics. Even if a motion model was used in the tracking algorithm, the accuracy of maximum velocity measurement that is limited by the available frame rate might not be fully compensated at the frame rate, 50 Hz, used in our study. Microbubble signals might be confused with the remaining tissue signals in the pixel intensity available from the commercial system. Ultrasound systems providing ultrafast technique and analytic signals/images can help prevent ULM technique from underestimating flow speeds. In addition, some diffuse and space-occupying lesions of the pancreas may cause changes in pancreatic microvessels, resulting in deviations in the evaluation of islet status. We admit that all animal models have certain limitations when simulating human diseases. The difference in β cell distribution and the number of microvessels between rodents and humans is worth considering.

## Conclusion

In conclusion, we have proposed a noninvasive in vivo microvascular imaging method that can be effectively used for visual and quantitative evaluation of disease progression and anti-cytokine immunotherapy efficacy in type 2 diabetes. We have elegantly demonstrated that ULM measurements were consistent with the various pathological changes in islet microvasculature and were highly correlated with β cell loss and deterioration of islet function. Moreover, ULM can supplement the lack of other clinical imaging examination methods, facilitate noninvasive monitoring of microvascular morphology, and visually evaluate the curative effect of anti-cytokine immunotherapy. This ULM technology based on clinical ultrasound equipment provides a direct evaluation method for the different microvascular-related diseases and can overcome the major limitations associated with lack of availability of β cell noninvasive disease course tracking and efficacy evaluation methods. Therefore, we are of the opinion that this technology holds the potential to establish a new standard for the clinical diagnosis and treatment of type 2 diabetes, offering benefits for patients.

## Data Availability

The data presented in this study are available on request from the corresponding author. The data are not publicly available due to the need for further analysis.
